# May Patients with Chronic Stroke Benefit from Robotic Gait Training with an End-Effector? A Case-Control Study

**DOI:** 10.3390/jfmk10020161

**Published:** 2025-05-06

**Authors:** Mirjam Bonanno, Paolo De Pasquale, Antonino Lombardo Facciale, Biagio Dauccio, Rosaria De Luca, Angelo Quartarone, Rocco Salvatore Calabrò

**Affiliations:** 1 IRCCS Centro Neurolesi Bonino-Pulejo, 98124 Messina, Italy; mirjam.bonanno@irccsme.it (M.B.); antonino.lombardo@irccsme.it (A.L.F.); biagio.dauccio@irccsme.it (B.D.); rosaria.deluca@irccsme.it (R.D.L.); angelo.quartarone@irccsme.it (A.Q.); roccos.calabro@irccsme.it (R.S.C.); 2 Department of Biomedical and Dental Sciences and Morphofunctional Imaging, University of Messina, 98125 Messina, Italy

**Keywords:** robotic gait training, neurorehabilitation, gait and balance, end-effector, lower limb, chronic stroke

## Abstract

**Background**: Gait and balance alterations in post-stroke patients are one of the most disabling symptoms that can persist in chronic stages of the disease. In this context, rehabilitation has the fundamental role of promoting functional recovery, mitigating gait and balance deficits, and preventing falling risk. Robotic end-effector devices, like the G-EO system (e.g., G-EO system, Reha Technology, Olten, Switzerland), can be a useful device to promote gait recovery in patients with chronic stroke. **Materials and Methods:** Twelve chronic stroke patients were enrolled and evaluated at baseline (T0) and at post-treatment (T1). These patients received forty sessions of robotic gait training (RGT) with the G-EO system (experimental group, EG), for eight weeks consecutively, in addition to standard rehabilitation therapy. The data of these subjects were compared with those coming from a sample of twelve individuals (control group, CG) matched for clinical and demographic features who underwent the same amount of conventional gait training (CGT), in addition to standard rehabilitation therapy. **Results:** All patients completed the trial, and none reported any side effects either during or following the training. The EG showed significant improvements in balance (*p* = 0.012) and gait (*p* = 0.004) functions measured with the Tinetti Scale (TS) after RGT. Both groups (EG and CG) showed significant improvement in functional independence (FIM, *p* < 0.001). The Fugl-Meyer Assessment—Lower Extremity (FMA-LE) showed significant improvements in motor function (*p* = 0.001, *p* = 0.031) and passive range of motion (*p* = 0.031) in EG. In EG, gait and balance improvements were influenced by session, age, gender, time since injury (TSI), cadence, and velocity (*p* < 0.05), while CG showed fewer significant effects, mainly for age, TSI, and session. EG showed significantly greater improvements than CG in balance (*p* = 0.003) and gait (*p* = 0.05) based on the TS. **Conclusions:** RGT with end-effectors, like the G-EO system, can be a valuable complementary treatment in neurorehabilitation, even for chronic stroke patients. Our findings suggest that RGT may improve gait, balance, and lower limb motor functions, enhancing motor control and coordination.

## 1. Introduction

Gait and balance alterations in post-stroke patients are some of the most disabling symptoms that can persist in the chronic stages of the disease [1,2,3]. Generally, post-stroke patients can manifest abnormal gait patterns characterized by temporal and spatial asymmetry between steps [4]. This asymmetry during gait could result in postural instability and a lack of coordination to both self-induced and external balance perturbations [5]. According to some authors [2,3], stroke-related gait deficits, leading to fall risk, comprise reduced propulsion at push-off, decreased hip and knee flexion during the swing phase, and reduced stability during the stance phase. All these gait features can represent the cause of falling in this patient population, leading to further medical complications. It is noteworthy that post-stroke patients represent a heterogeneous group due to variations in the location and extent of brain damage. As a result, they may exhibit diverse step length patterns in both the paretic and non-paretic limbs. These variations are often linked to compensatory adjustments involving the pelvis and the unaffected side of the body [6].

In this context, physiotherapy is fundamental to promoting functional recovery, mitigating gait and balance deficits, and preventing fall risk [7,8]. Both conventional rehabilitation approaches and innovative technologies have proved their effectiveness in post-stroke patients [7,8]. However, limited evidence exists in the context of chronic stroke patients [9,10], in which it is commonly thought that neuroplastic processes and the subsequent functional recovery are already exhausted.

Among the innovative approaches, robotic rehabilitation devices play a crucial role, enabling intensive, repetitive, and task-oriented training to stimulate neuroplastic processes, even in chronic stages [10,11]. Robotic devices are generally categorized into two main types based on their biomechanical interaction with patients: exoskeletons and end-effectors [8].

End-effectors, such as the G-EO system (Reha Technology, Olten, Switzerland), are equipped with a single distal control, providing a propulsive motion of the legs thanks to two footplates that replicate the motion of stance and swing phases during gait training [12]. This design contrasts with exoskeletons, wearable powered orthoses that guide joint movements more proximally (e.g., Ekso-GT, Indego, or Lokomat) [12,13]. While exoskeletons, especially the Lokomat, have demonstrated effectiveness in promoting functional recovery and brain connectivity changes in chronic post-stroke patients [10], evidence supporting the efficacy of end-effector devices, particularly the G-EO system, remains limited [14] in this population. Most available studies have focused on acute and subacute stages [15,16,17], leaving a gap in the literature regarding their use in chronic stroke.

Given this background, this study aims to evaluate the effects of robotic end-effector gait rehabilitation training (RGT) in patients with chronic stroke and compare the functional outcomes with a matched control group receiving conventional gait training (CGT). To achieve this, we clinically assessed both rehabilitation approaches at baseline and post-treatment. A linear mixed effects (LME) model was employed to analyze the influence of demographic, clinical, and rehabilitation variables on the outcomes in both groups.

## 2. Materials and Methods

Twelve chronic post-stroke patients, attending the Robotic and Behavioral Neurorehabilitation Unit of the IRCCS Centro Neurolesi “Bonino-Pulejo” between January 2021 and August 2021, were evaluated for inclusion in this study.

Inclusion criteria were (i) hemiplegia/hemiparesis due to a first-ever stroke; (ii) at least one year after stroke; (iii) supervision-dependent ambulation (Functional Ambulation Classification—FAC ≥ 3); (iv) patients aged between 18 and 75 years. Patients were excluded if they presented the following criteria: (i) age > 75 years; (ii) diagnosis of concurrent psychiatric conditions or other significant medical comorbidities that could interfere with the RGT; (iii) presence of deficits (e.g., cognitive, visual, or auditory) that could limit the comprehension and/or execution of the proposed exercise; (iv) comorbidities that prevented upright posture and walking (e.g., hypotension); (v) refused consent or were unable to provide informed consent; (vi) recent bone fractures. In addition, we excluded patients if they had contraindications to the use of the technological instrumentation, such as a weight > 150 kg and open lesions or bandages in contact with the harness.

The enrolled patients were evaluated at baseline (T0) and after the experimental training (T1), consisting of forty sessions of RGT (experimental group—EG) for eight consecutive weeks.

The data of these subjects were compared with those coming from a sample of 12 individuals (control group, CG) matched for clinical and demographic features who underwent the same amount of CGT with no robotic assistance, in addition to standard rehabilitation therapy.

All experiments were conducted according to the ethical policies and procedures approved by the local ethics committee (IRCCSME-45/2020 approved on 14/12/2020). All participants gave their written informed consent.

The clinical–demographic characteristics of both groups are summarized in Table 1.

### 2.1. Procedures

All the included patients were submitted for the same amount of training. However, they diverged in the type of rehabilitation approaches. The EG (n. 12) underwent RGT with the G-EO system, while the CG (n. 12) received CGT. Both groups followed the same rehabilitation protocol (as per the total amount of training), consisting of five sessions per week over eight weeks (by our established clinical research and standard care protocols). Each session included approximately one hour of gait training, followed by 45 min of conventional physiotherapy. Importantly, while the duration and structure of the sessions were identical, the type of gait training differed between groups: the EG used the G-EO system, whereas the CG received conventional overground gait training. In addition to gait training, both groups also received conventional physiotherapy, including stretching exercises, passive and active-assisted limb mobilization, and therapeutic exercises aimed at improving range of motion and overall muscle strength, lasting approximately 45 min per session (see Table 2 for more details).

All patients underwent a clinical visit and neurological evaluation at T0 and T1. However, only the patients who underwent RGT were also evaluated with the Fugl-Meyer—Lower Extremity assessment to understand the effects of robotics on body segmental outcomes, as well as specific parameters coming from the device.

### 2.2. Outcome Measures

Motor and functional clinical assessment scales/tests were administered by a skilled physiotherapist (ALF) at T0 and T1, and the subsequent analyses were performed by a biomedical engineer (PDP). In particular, the physiotherapist, who was blinded to the training allocation of the patient, administered a complete motor battery, including (i) the 10 Meters Walking Test (10MWT) [18] to assess walking speed in meters per second over a short distance; (ii) the Tinetti Scale (TS) [19], made up of 16 items, with 7 items assessing gait (with scores ranging from 0 to 12) and 9 items evaluating balance (with scores ranging from 0 to 16); (iii) the Modified Ashworth Scale (MAS) [20] was used to assess spasticity in lower limbs (hip, knee, and ankle), and it ranges from 0 (no increase in muscle tone) to 4 (rigid limb movement). Intermediate scores indicate varying degrees of increased muscle tone, from slight resistance (1, 1+) to marked stiffness (2, 3); (iv) the Functional Independence Measure (FIM) [21] was utilized to assess overall functioning across 18 items divided into six subscales—self-care, sphincter control, transfer, locomotion, communication, and social cognition ability. A higher score indicates less disability for basic daily functions.

After the allocation assignment, EG patients were also evaluated with the Fugl-Meyer—Lower Extremity (FMA-LE) assessment [22], which assesses reflexes and motor functioning at the hip, knee, and ankle (part E ranging from 0 to 28), coordination (part F ranging from 0 to 6), sensory functioning (part H ranging from 0 to 12), joint range of motion (part JI ranging from 0 to 20), and joint pain (part JII ranging from 0 to 20). In addition, specific rehabilitation parameters, regarding RGT, were extracted from the G-EO system, such as mean values of step cadence (steps/min) and walking speed (m/s).

### 2.3. G-EO System

The G-EO system [23] is an advanced robotic end-effector designed specifically for lower limb rehabilitation. This innovative device is equipped with footplates, or pedals, that guide the movement of the lower limbs along fully programmable trajectories. These footplates are designed to facilitate a range of natural motion patterns, moving from the bottom to the top in a manner that closely mimics real-life walking. The system allows for precise adjustment of key parameters, including step length (from 0 to 55 cm), step height (up to 400 mm), footplate angles (up to 90 degrees), movement velocity (up to 2.3 km/h), and acceleration peaks (up to 10 m/s^2^). These customizable settings enable tailored rehabilitation programs that meet individual patient needs.

Unlike traditional systems that rely on the hips and knees for leg movement, the G-EO system focuses on ankle actuation, which is critical for simulating accurate walking mechanics. The patient’s feet are securely fastened to the footplates using straps, ensuring proper alignment and safety during therapy sessions. This setup enables the device to replicate forward and backward walking motions, as well as stair-climbing activities, including both ascending and descending movements.

To enhance patient safety and support, the system features handrails on both sides, providing stability during therapy. Additionally, it incorporates a Body Weight Support (BWS) system, which reduces the load on the patient’s lower limbs and allows for controlled weight-bearing exercises. This combination of features makes the G-EO system a highly versatile and effective tool for gait rehabilitation, offering a comprehensive approach to improving mobility and function in patients with lower limb impairments. The gait training session can be monitored through two displays providing visual feedback for therapists and patients. Moreover, this robotic device allows clinicians to supervise therapy progress over time through a detailed report, in which mean values of step length and cadence, walking speed, number of steps, and stairs are reported. In this way, therapists and clinicians can personalize each gait training session according to patients’ parameters. During the rehabilitation sessions conducted on the G-EO system, patients began walking at an initial comfortable pace. This velocity was incrementally increased by 0.1 m/s based on the motor capacity and the state of fatigability of the patients. Once this threshold was achieved, the main phase of the session began. This progressive approach was implemented daily, with adjustments made based on the patient’s performance and safety. Additionally, the therapists were allowed to modify step length parameters to tailor the RGT session to each patient. The primary goal was to increase gait parameters such as step length and velocity within safe and effective ranges. If a patient demonstrated the ability to sustain these parameters, those parameters were retained for the session. If not, the training proceeded at the previously established parameters from the prior session. The intensity of the training was individualized to prevent overexertion, with patients being closely monitored by clinicians. In instances of noticeable fatigue, the walking speed was decreased to a more comfortable pace, ensuring the patient could continue training effectively without risking overfatigue or injury. In general, all the RGT sessions were supervised by the physiotherapist, providing support to the paretic knee of the patient as needed.

**Figure 1:**
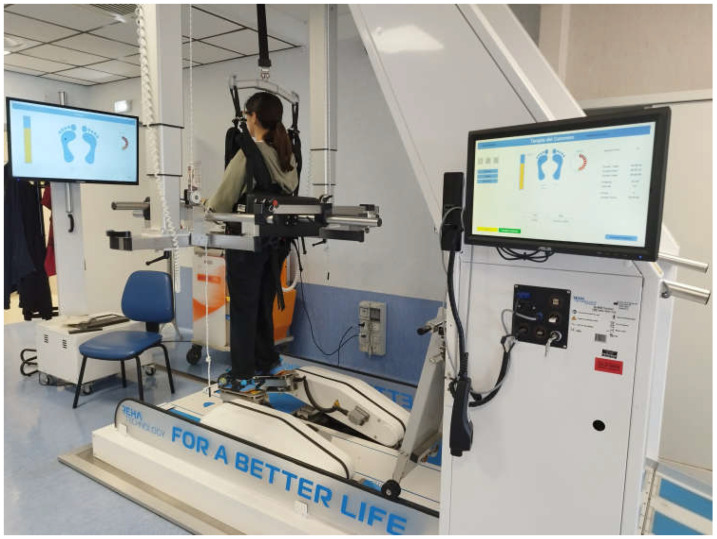
Post-stroke patient performing gait training on the G-EO system. The figure shows a patient performing an RGT session on the G-EO system. The monitor in front of the patient, as well as the second monitor of the therapist, provides visual real-time feedback on the patient’s performance, including footplate pressure feedback, active therapy duration, repetitions, and distance covered.

Initially, BWS was set at 80% discharge for both floor walking and stair climbing, gradually decreasing by 10% each week until reaching 10% or the highest tolerated BWS; however, individual adjustments were implemented as needed based on patient tolerance and fatigue to ensure a safe and effective progression. Refer to Figure 1 for a visual representation.

### 2.4. Conventional Gait Training

Conventional gait training (CGT) sessions lasted one hour, followed by 45 min of conventional physiotherapy, in line with the protocol used for the EG (see Table 2).

In particular, the CGT included various exercises such as weight-shifting, core muscle strengthening, monopodal and bipodal balance exercises, and gait training involving obstacles, tandem, and slalom walking.

Like the EG, the intensity of training in the CG was individually tailored to prevent fatigue, with close monitoring of patients. Additionally, throughout all sessions, post-stroke patients received manual guidance and supervision from physiotherapists to minimize the risk of falls.

### 2.5. Statistical Analysis

The sample participants’ sociodemographic and clinical parameters were analyzed, and differences between the experimental and control groups were assessed. Descriptive statistics were expressed as mean ± standard deviation for continuous variables and as frequencies (percentages) for categorical variables. Age, years of education, and years since injury were treated as continuous variables, while gender, etiology, and most affected side were analyzed as categorical variables. Normality for continuous variables was tested using the Shapiro–Wilk test (MATLAB R2022a, Natick, MA, USA, function swtest). Because the age distribution in both groups was normal, a parametric *t*-test (MATLAB function ttest) was used to evaluate group differences for age. In contrast, due to non-normal distributions of years of education and years since injury, group differences for these variables were assessed with the Mann–Whitney U test (MATLAB function ranksum). For the categorical variables, contingency tables were constructed; Fisher’s exact test (MATLAB function fishertest) was applied when expected frequencies were low, whereas a chi-squared test (MATLAB function chi2cdf) was used otherwise.

Clinical data collected before and after treatment were compared within both groups. First, normality was evaluated using the Shapiro–Wilk test, and because not all distributions were normal, non-parametric methods were employed. Within-group changes were assessed with the Wilcoxon signed-rank test on paired pre-treatment (T0) and post-treatment (T1) data. To evaluate differences in treatment effects between groups, we computed the change (delta) for each subject (post-treatment value minus pre-treatment value) and compared these delta values between the experimental and control groups using the Mann–Whitney U test (i.e., a rank-sum test on the pre–post differences). Baseline equivalence was further confirmed by comparing the T0 values of the experimental group with those of the control group using the same non-parametric approach.

The dependency of clinical evaluations on demographic factors and patients’ clinical status was tested using linear mixed effects models (LMEs) [24] via MATLAB’s fitlme function. Separate models were constructed for the experimental and control groups, with participants included as a random effect to account for inter-individual variability. The age (A), gender (G), time since injury (I), and session (S) were treated as fixed effect factors. The experimental factors G and S were treated with categorical (dummy) variables, as they could take categorical outcomes: G = 0 for male, G = 1 for female, S = 0 for T0, S = 1 for T1. RGT parameters, which were selected by the therapist as step cadence (C) and velocity (V) during therapy, were included in the EG model based on their established relevance as indicators of gait function and rehabilitation outcomes after stroke [25,26,27]. Moreover, the interactions between sessions and each variable were evaluated. The normality of the residuals, which supports the model choice, was verified using a Lilliefors test applied to the residuals.

(1) $$Y={g(u}_{0}+\alpha_{0}A+\beta_{0}G+\gamma_{0}I+\delta_{0}S+\theta_{0}SA+\iota_{0}SG+\kappa_{0}SI+\epsilon )$$

(2) $$Y={g(u}_{0}+\alpha_{0}A+\beta_{0}G+\gamma_{0}I+\delta_{0}S+\zeta_{0}C+\eta_{0}V+\theta_{0}SA+\iota_{0}SG+\kappa_{0}SI+\lambda_{0}SC+\mu_{0}SV+\epsilon )$$

**Equations.** In Equations (1) and (2), u_0_ represents the individual intercept and accounts for interindividual differences. The coefficients α_0_, β_0_, γ_0_, δ_0_, ζ_0_, η_0_, θ_0_, ι_0_, κ_0_, λ_0_, and μ_0_ represent fixed effects; thus, the modulation of the response variable by the main factors A, G, I, S, C, V, and their interactions, ϵ, is the error-term.

In both Equations (1) and (2), g represents the identity link function. Control data were fitted using Equation (1), while experimental data were fitted with Equation (2), which considers rehabilitation training input parameters. In both cases, model parameters were estimated using maximum likelihood.

The significance of each fixed-effect term in the models was tested using the Wald Test [24], which evaluates the contribution of each fixed effect based on its coefficients and associated covariance structure. Given the number of clinical outcome measures and fixed effects analyzed, the risk of inflated type I errors due to multiple comparisons was addressed by applying a False Discovery Rate (FDR) correction using the Benjamini–Hochberg procedure [28].

The FDR correction was applied to the *p*-values obtained from the Wald tests at the term level, separately within each outcome-specific model. This correction method was applied independently for each clinical outcome, reflecting the independent testing of distinct functional domains.

Both uncorrected and FDR-corrected *p*-values are provided in the Supplementary Materials (Tables S1 and S2). Statistical significance was based on FDR-corrected *p*-values; effects not surviving correction but with *p*-values close to the threshold were interpreted cautiously as indicative of trends.

## 3. Results

At the baseline, there were no significant differences in the demographic and clinical features between the groups, as shown in Table 1. All patients completed the trial, and none reported any side effects during or after the training.

We found that the TS evaluating balance and gait was significantly improved after (T1) the RGT in the EG (*p* = 0.012, *p* = 0.004). Regarding the 10MWT, we found a statistically significant improvement in the CG (*p* = 0.014), while the EG showed an increased score, without achieving statistical significance. Moreover, we found that independence in daily activities (FIM) in both groups (*p* < 0.001), as shown in Table 3.

**Figure 2:**
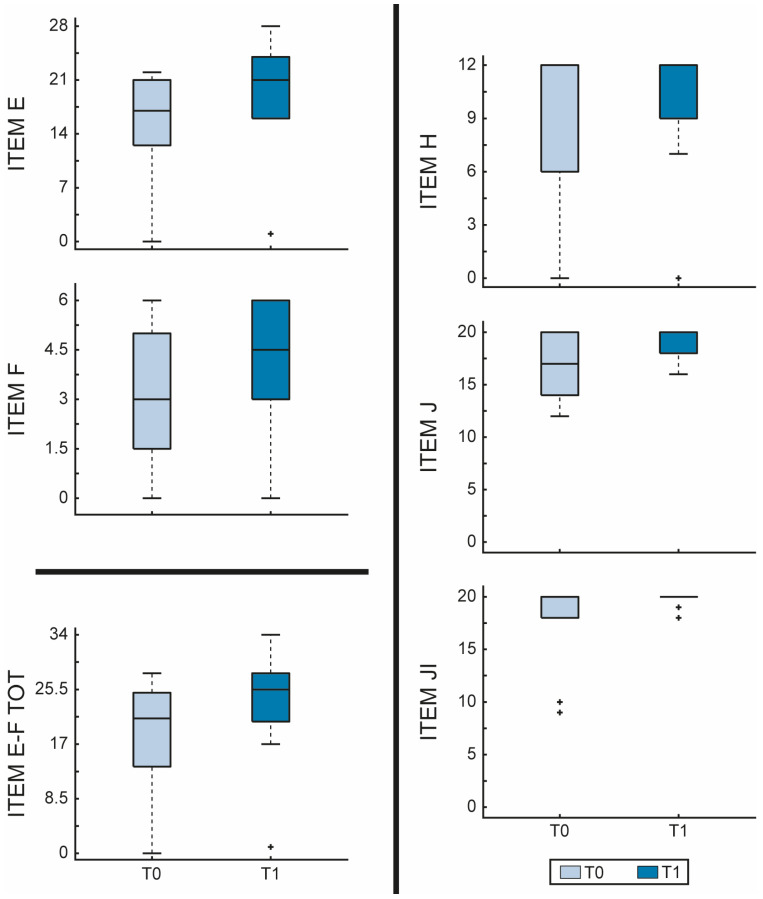
Fugl-Meyer Assessment—Lower extremity (FMA-LE) scores evaluated before and after RGT in the EG. The figure shows boxplots for each item of FMA-LE comparing before (T0—light blue) and after (T1—dark blue) RGT. Item E = lower extremity; Item F = coordination/speed; Item total E-F = motor function; Item H = sensation; Item JI = passive joint motion; Item JII = joint pain. The outliers are plotted using the + marker symbol.

On the other hand, the FMA scores also improved, especially in the motor function (items E-F) (E, *p* = 0.001, F, *p* = 0.031, E-F, *p* = 0.001) and in the passive range of motion (JI, *p* = 0.031), as shown in Figure 2.

At the baseline, there were no significant differences in the clinical scales/tests scores between the two groups, as shown in Table 3. Regarding the statistical comparison between the two groups, we found statistically significant differences between EG and CG only in the TS for balance and gait (*p* = 0.003; *p* = 0.05), as shown in Table 3.

**Figure 3:**
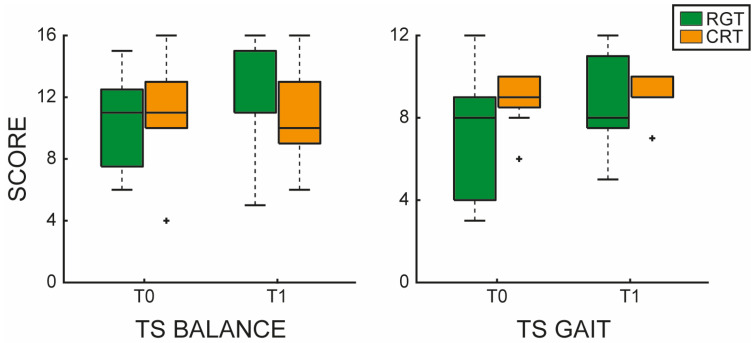
Tinetti Scale (TS) evaluating balance and gait functions. The left panel shows the TS balance boxplot comparing before (T0) and after (T1) RGT (green) and CGT (orange). The right panel shows the TS gait boxplot comparing before (T0) and after (T1) RGT (green) and CGT (orange). The outliers are plotted using the + marker symbol.

In particular, the EG showed a greater improvement in the TS for both balance and gait, compared to the CG, as shown in Figure 3.

The analysis, conducted using an LME model and evaluated via the Wald Test, was performed to detect treatment-associated changes while simultaneously assessing the influence of various factors (age, gender, TSI, and session) on clinical outcomes. Additionally, for the experimental group, treatment-specific parameters, such as velocity and cadence, which were adjusted by the therapist session by session, were integrated into the model. This comprehensive approach enabled us to identify the parameters that most significantly influenced the success of the intervention, thereby providing valuable insights into the determinants of treatment efficacy. In this study, we have reported only the statistically significant effects detected by the model.

In the TS evaluating balance in the EG (R^2^ = 0.98), significant effects were found for velocity (*p* = 0.049), gender × session (*p* = 0.049), TSI × session (*p* = 0.016), cadence × session (*p* = 0.016), and velocity × session (*p* = 0.016). Also, for the TS evaluating gait in the EG (R^2^ = 0.98), significant effects were detected for session (*p* < 0.001), velocity (*p* < 0.001), gender × session (*p* < 0.001), and velocity × session (*p* < 0.001). In the control group (R^2^ = 0.83), significant interactions were found for age × session (*p* = 0.019) and TSI × session (*p* = 0.024). For the MAS Scale in the EG (R^2^ = 0.97), significant effects were observed for gender (*p* = 0.020), while a trend toward a significant effect of TSI (*p* = 0.074) was observed. Regarding the FIM Scale in the EG (R^2^ = 0.98), we found that significant effects were observed for gender (*p* = 0.001), TSI (*p* < 0.001), cadence (*p* < 0.001), velocity (*p* =0.024), age × session (*p* = 0.024), gender × session (*p* < 0.001), and cadence × session (*p* < 0.001). On the other hand, in the CG (R^2^ = 0.96), we found only the following significant effects: session (*p* = 0.023), TSI (*p* = 0.023), while a trend toward a significant effect of the interaction gender × session (*p* = 0.065) was observed. For FMA Item H in the EG (R^2^ = 1), significant effects were detected for TSI (*p* = 0.019), velocity (*p* < 0.001), age × session (*p* < 0.001), and TSI × session (*p* < 0.001). For Item JI in the experimental group (R^2^ = 0.97), significant effects were found for session (*p* < 0.001), TSI (*p* = 0.013), age × session (*p* < 0.001), gender × session (*p* < 0.001), TSI × session (*p* = 0.012), and a trend toward a significant effect of the interaction velocity × session (*p* = 0.079) was observed. For Item JII in the experimental group (R^2^ = 0.93), significant effects were observed for TSI (*p* < 0.001), cadence (*p* = 0.017), and TSI × session (*p* < 0.001).

Although the effect on the 10MWT evaluation in the EG (R^2^ = 0.99) did not reach statistical significance after FDR correction, a trend toward significance was observed for the following interactions: gender × session (*p* = 0.073), TSI × session (*p* = 0.089), and cadence × session (*p* = 0.073).

## 4. Discussion

In this case-control study, we aimed to investigate the effects of rehabilitation with the G-EO system, a robotic end-effector, on lower limb function in chronic post-stroke patients, compared to a CG that received CGT. At baseline, no significant differences were observed between groups in demographic and clinical characteristics. However, following the intervention, the EG demonstrated significant improvements in balance and gait performance (TS evaluating gait and balance, see Figure 3), as well as significant differences compared to the CG. These results may indicate that RGT enhanced postural control and dynamic stability, likely due to its ability to provide task-specific and repetitive movement patterns, which might facilitate neuromuscular adaptation (as demonstrated by previous studies [29,30]). In line with our findings, Kim et al. [31] found that RGT with an end-effector, combined with conventional physiotherapy, was associated with an improvement in balance scores, in comparison with CGT alone. According to the literature [2,5,32,33], lower limb alterations (e.g., muscle stiffness, spasticity, sensory loss) in post-stroke patients can be associated with a reduced efficiency in gait and balance, leading to an increased risk of falls in this patient population. In this context, neurorehabilitation strategies aim to achieve better neuromuscular control of lower limbs to promote a higher level of independence [34]. Innovative technologies, like robotic devices, have the advantage of carrying out personalized and task-oriented training that can motivate the patients, leading to longer therapy sessions [8]. Specifically, end-effector robotic devices are stationary devices with a distal movable part requiring active participation. In particular, the end-effectors are suitable tools that can be adapted to different shapes and sizes. Our results are likely influenced by the suitability of end-effector devices for patients who had residual locomotor functions, indicating a sufficient activation of proximal joints and muscles. It is noteworthy that the G-EO system provides repetitive, intensive, and task-oriented training that can enhance motor learning by engaging both efferent motor pathways and afferent sensory pathways throughout the training process [35]. Repetitive movements guided by the robotic device can play a crucial role in the re-acquisition of motor functions, promoting muscle memory and improving motor coordination [35]. It has been demonstrated that repetitive motor tasks facilitate neuroplasticity and brain reorganization in stroke patients, resulting in enhanced motor recovery after stroke [36]. In addition, the G-EO system promotes proximal muscle activity. In this sense, some authors found that proximal lower limb control plays a key role in improving gait speed and walking performance after stroke [36]. In this regard, we found that the RGT session with the G-EO system influenced the 10MWT scores in the EG. However, we did not find statistically significant differences between CG and EG in gait speed, maybe due to the small sample. Moreover, other authors studied the effects of RGT in chronic stroke patients with Lokomat, an exoskeleton, suggesting that FMA scores were significantly different in comparison with the conventional rehabilitation group. However, no significant differences in other clinical scales or tests were found in both groups. According to our results, RGT with the G-EO system improved FMA-LE scores, especially lower limb movements (FMA-LE part E), coordination (FMA-LE part F), and joint passive range of motion (FMA-LE part JI). This aspect could be explained by the fact that repetitive movements, provided by the robotic device, can serve as a fundamental element in the acquisition of motor skills [37], improving motor coordination and precision over time. A key outcome of this study was the improvement in functional independence (FIM) in both groups. This suggests that while both RGT and conventional therapy contribute to functional recovery, RGT with the G-EO system may offer additional benefits, allowing for the simulation of real-world experiences, such as ascending and descending stairs. In this way, by incorporating different stimuli and environmental conditions, therapy sessions become more dynamic and reflect the challenges encountered in everyday life [38], explaining our improvements in FIM scores.

The LME analysis showed that RGT treatment parameters (e.g., velocity and cadence) had significant effects across different assessment scales, indicating that adjustments made during the intervention have a dynamic impact on motor function recovery. In addition, as the LME analysis revealed that patients’ demographic and clinical characteristics influenced various assessment scales, these findings underscore the importance of adopting a personalized rehabilitation approach that considers both baseline characteristics and adaptive treatment strategies to optimize motor recovery. Moreover, some clinical scales, such as FMA, showed significant interactions between sessions and various fixed effects. For instance, we found a significant session × age interaction (FMA, items H and JI), suggesting that the magnitude or pattern of motor recovery differs across age groups, with younger patients potentially exhibiting a more pronounced improvement than older patients.

This aspect is particularly important, as in the acute and subacute phases of stroke, it appears that there is no significant difference in motor and cognitive recovery between younger and older patients. Several studies, including the one by Kugler et al. [39], have demonstrated that age is a poor predictor of functional recovery during these early stages. However, in the chronic phase of stroke, age seems to play a more substantial role. Yoo et al. [40] found that functional recovery between 6- and 30-months post-stroke differed significantly between patients younger than 70 years and those aged 70 years or older.

Moreover, we found a statistically significant effect of velocity and an interaction between velocity and session on gait and balance outcomes (TS), as well as the effect of the interaction between cadence and session on balance outcome (TS for balance). According to Tomida et al. [41], increasing walking speed on a treadmill can significantly increase cadence in post-stroke patients. Generally, in healthy individuals, increasing gait speed requires taking longer steps, which necessitates moving the leg forward more quickly [42]. On the other hand, in post-stroke patients, enhancing propulsive force and facilitating a rapid forward swing of the lower limbs, achieved through an increased push-off moment via ankle plantar flexion during the late stance phase, augmented pull-off via hip flexion in the initial swing phase, and greater angular velocity, are key factors in improving walking speed [41].

Walking with end-effectors allows high degrees of freedom motions, which may provide a more realistic gait [43]. This aspect could have increased the passive range of motion scores in FMA-LE (FMA item JI), suggesting that the G-EO promoted an effective recovery instead of relying solely on behavioral compensation mechanisms. Furthermore, the significant effects and potential trends identified in passive range of motion (FMA item JI) and joint pain (FMA item JII) highlight the complex interplay between intrinsic patient characteristics and the optimization of tailored treatment parameters.

Unlike conventional rehabilitation training, the RGT with the G-EO offers visual feedback for patients and therapists. In this way, patients could have exploited the visual cues on the screen to increase their motivation during the training session. According to Nascimento et al. [44], walking training accompanied by cues for cadence can be an effective rehabilitation strategy to positively affect cadence, speed, and step length. On the other hand, the 10MWT showed a significant improvement in the CG, while the EG exhibited an increased score without reaching statistical significance. This finding suggests that CGT may still be more effective for certain aspects of mobility.

Similar findings were reported by Goffredo et al. [45], where the 10MWT did not show significant improvement in the RGT group treated either with an end-effector or with an exoskeleton, while the CGT group achieved statistical significance in this test. Likewise, in the study by Aprile et al. [14], the 10MWT did not yield any significant differences between T0 and T1 in either the RGT or CGT groups.

However, in our study, the LME analysis revealed a potential trend of the following interactions: age × session, gender × session, TSI × session, and cadence × session in the EG, related to the 10MWT, indicating that considering both demographic and clinical patients characteristics as well as RGT training parameters can positively affect the outcomes over time.

Among the interactions identified in the 10MWT evaluation for the EG, the cadence × session is particularly noteworthy. This finding potentially indicates that changes in cadence throughout the treatment could be significantly associated with improvements in walking performance [44]. Given that cadence represents the rhythm and pace of steps [46], a critical element of effective gait, the significant interaction suggests that adjustments made to cadence during each session contributed meaningfully to overall performance gains [47]. Specifically, the dynamic modulation of cadence, which was tailored by the therapist on a session-by-session basis, appears to have played a crucial role in enhancing neuromuscular coordination and gait efficiency. As the treatment progressed, the evolving relationship between cadence and session highlighted its potential as both a therapeutic target and a sensitive indicator of treatment responsiveness. In essence, these results underscore the importance of personalized adjustments in cadence to optimize rehabilitation outcomes, suggesting that fine-tuning this parameter could be key to achieving more effective gait training in patients undergoing robotic-assisted interventions [48,49].

Regarding muscle tone and motor control, both the EG and CG exhibited lower MAS scores post-treatment compared to baseline, indicating a reduction in spasticity. However, while the overall improvement in MAS scores was not statistically significant in the CG, the EG demonstrated statistically significant effects of gender and a trend toward a significant effect of TSI.

These findings suggest that, although both interventions led to a decrease in muscle tone, RGT may have a more pronounced impact on reducing spasticity in certain subgroups of patients [15]. In particular, the effect of gender and TSI can modulate the response of the MAS to robotic-assisted therapy, underscoring the potential for a more patient-specific rehabilitation approach.

According to a previous study in the multiple sclerosis population [35], the RGT with the G-EO system showed that MAS scores were significantly improved compared to the CGT. It has been suggested that RGT, by stimulating neuroplastic processes, may help regulate corticospinal excitability mechanisms involved in spasticity [8,35]. Spasticity is characterized by an increase in velocity-dependent tonic stretch reflexes, often associated with exaggerated tendon jerks [50]. From this perspective, the repetitive movements performed at a tolerated speed, according to the patients’ needs, during RGT could mitigate spasticity by engaging spinal locomotor centers and influencing corticospinal pathway activity.

Our study has some limitations that should be acknowledged. First, the main limitation is related to the lack of randomization. Randomized trials (RCTs) allow for an unbiased assessment of treatment effects and ensure that, on average, treatment groups are balanced across all covariates. Secondly, the small sample prevents us from generalizing our findings. In this context, future larger sample RCTs on RGT in chronic post-stroke patients should be designed to minimize selection bias. Increasing the sample size would enhance the statistical power to confirm the positive trend of our study. Furthermore, we employed a demographic matching approach in our case-control design, which offers more limited control over potential confounding factors. Unlike simple demographic matching, propensity score matching accounts for multiple covariates simultaneously to achieve a more comprehensive balance between groups. In our study, the two groups were mostly balanced, although the TS gait at baseline was close to reaching statistical significance. Therefore, future studies should aim for greater homogeneity between groups to improve comparability and reduce potential confounding effects.

Future research should incorporate quantitative gait analysis methods, such as motion capture systems, force plates, and electromyography, to provide deeper insights into the effects and underlying mechanisms of motor recovery during RGT in chronic stroke patients. It is also important to implement clinical and quantitative evaluations not only in the short term but also in the long term through appropriate follow-up assessments. Lastly, we cannot exclude the possibility that the RGT group experienced a psychological placebo effect as compared to the non-robotic group, potentially influencing the results. However, unlike pharmacological or neuromodulation studies, where sham treatments can be implemented to control placebo effects, rehabilitation research faces inherent limitations: patients are fully aware of the type of intervention they receive, making it difficult to eliminate such biases.

Our study was intended to be preliminary evidence focusing on the feasibility and potential effectiveness of RGT with an end-effector in post-stroke patients compared to conventional gait training to provide basic information on determining the appropriateness of candidates that could benefit from this kind of treatment.

## 5. Conclusions

In conclusion, our results suggest that RGT with end-effectors, such as the G-EO system, may serve as an adjunctive and complementary treatment in neurorehabilitation, even for patients with chronic stroke, where recovery is typically considered to have plateaued. According to our findings, the G-EO system improved gait and balance functions compared to CGT. Furthermore, RGT promoted improvements in lower limb motor functions, indicating enhanced motor control and coordination, which are both key factors in fall prevention.

## Figures and Tables

**Table 1:** Demographic and clinical characteristics of both groups (experimental—RGT and control—conventional rehabilitation training).

Characteristic	All	EG	CG	*p* -Value	Test
**Patients**	24 (100%)	12 (50%)	12 (50%)		
**Age**	51.13 ± 13.72	47 ± 12.54	55.25 ± 14.12	0.14	*t*-test
**Education (years)**	12.92 ± 2.96	12.92 ± 2.64	12.92 ± 3.37	0.82	U
**Sex**					
** Male**	16 (66.67%)	8 (66.67%)	8 (66.67%)	1	Fisher
** Female**	8 (33.33%)	4 (33.33%)	4 (33.33%)	1	Fisher
**Time since injury (years)**	2.25 ± 1.62	2.33 ± 1.78	2.17 ± 1.53	1	U
**Etiology**					
** Ischemic**	13 (54.17%)	7 (58.33%)	6 (50%)	0.68	χ^2^
** Hemorrhagic**	11 (45.83%)	5 (41.67%)	6 (50%)	0.68	χ^2^
**Most affected side**					
** Left**	11 (45.83%)	5 (41.67%)	6 (50%)	0.68	χ^2^
** Right**	13 (54.17%)	7 (58.33%)	6 (50%)	0.68	χ^2^

**Legend:** Fisher (Fisher’s exact test); χ^2^ (chi-squared test); U (Mann–Whitney U test).

**Table 2:** Summary of the characteristics linked to RGT and CGT protocols.

Rehabilitation Intervention Elements	CGT	RGT
Setting	Conventional physiotherapy gym	G-EO system robotic device
Duration of gait training	1 h	1 h
Gait training	10′ postural alignment with the aid of the physiotherapist 20′ gait training exercises: walking over obstacles of different sizes, tandem and slalom walking 10′ weight-shifting, core muscle strengthening, monopodal and bipodal balance exercises, 10′ ascending and descending stairs 10′ cool down and return to sitting position	10′ postural alignment with G-EO system 30′ gait training on the G-EO system, focusing on proper weight distribution between the two legs 10′ ascending and descending stairs with G-EO 10′ cool down and return to sitting position
Ascending and descending stairs	Yes With the supervision and manual guidance of the physiotherapist	Yes With the supervision of the physiotherapist and the guidance provided by the robotic device
Guidance	Manual guidance provided by the physiotherapist	BWS and robotic guidance—when needed, the physiotherapist provided supervision and/or hands-on support to the paretic knee of the patients
Audio–visual Feedback	Verbal feedback provided by the physiotherapist	Visual feedback provided by the monitor of the G-EO system and verbal feedback provided by the physiotherapist, as needed
Fatigue management	Yes Visible signs of fatigue (e.g., changes in gait quality or shortness of breath)	Yes Visible signs of fatigue (e.g., changes in gait quality or shortness of breath)
Rehabilitation parameters	No	Yes Step cadence and velocity
Conventional physiotherapy	Yes 45′ of stretching exercises, passive and active-assisted limb mobilization, and therapeutic exercises	Yes 45′ of stretching exercises, passive and active-assisted limb mobilization, and therapeutic exercises

**Table 3:** Statistical comparisons (*p*-values) between T0 and T1 for each group and between EG and CG at T0 and T1 (EG vs. CT).

Scale/Test	EG T0–T1	CG T0–T1	EG vs. CG T0–T0	EG vs. CG Δ (T1–T0)
**10MWT**	0.069	**0.014**	0.295	0.255
**Tinetti–Balance**	**0.012**	0.277	0.6	**0.003**
**Tinetti–Gait**	**0.004**	0.25	0.06	**0.05**
**MAS**	0.078	0.453	0.17	0.342
**FIM**	**<0.001**	**<0.001**	0.977	0.183

**Legend:** 10WMT (10 m Walking Test), MAS (Modified Ashworth Scale), FIM (Functional Independence Measure); Δ (T1–T0): difference of “after treatment–before treatment”. Significant *p*-values are reported in bold.

## Data Availability

The data presented in this study are available on request from the corresponding author. The data are not publicly available due to the privacy of research participants.

## Supplementary Materials

The following supporting information can be downloaded at https://www.mdpi.com/article/10.3390/jfmk10020161/s1, Table S1: Results of linear mixed effects models for the experimental group (RGT): corrected and uncorrected *p*-values and significance for each clinical outcome. The table reports the results of linear mixed effects models (LMEs) for each clinical outcome in the experimental group. For each model, *p*-values obtained from Wald tests at the term level and their corresponding false discovery rate (FDR)-corrected values are presented, along with the assigned significance symbols. Statistical significance is indicated as *** *p* < 0.001, ** *p* < 0.01, * *p* < 0.05 after FDR correction, † indicates results significant before but not after correction, ns denotes non-significant effects; Table S2: Results of linear mixed effects models for the control group (CGT): corrected and uncorrected *p*-values and significance for each clinical outcome. The table reports the results of linear mixed effects models (LMEs) for each clinical outcome in the experimental group. For each model, *p*-values obtained from Wald tests at the term level and their corresponding false discovery rate (FDR)-corrected values are presented, along with the assigned significance symbols. Statistical significance is indicated as *** *p* < 0.001, ** *p* < 0.01, * *p* < 0.05 after FDR correction, † indicates results significant before but not after correction, ns denotes non-significant effects.

## Abbreviations

The following abbreviations are used in this manuscript:

10MWT	10 Meters Walking Test
BWS	Body Weight Support
CG	Control Group
CGT	Conventional Gait Training
EG	Experimental Group
FAC	Functional Ambulation Classification
FDR	False Discovery Rate
FIM	Functional Independence Measure
FMA-LE	Fugl-Meyer Assessment—Lower Extremity
LME	Linear Mixed Effects
MAS	Modified Ashworth Scale
RGT	Robotic Gait Training
T0	Baseline Timepoint
T1	Post-Treatment Timepoint
TS	Tinetti Scale
TSI	Time Since Injury
