# Supplementary material for: May Patients with Chronic Stroke Benefit from Robotic Gait Training with an End-Effector? A Case-Control Study
Source: J Funct Morphol Kinesiol. 2025 May 6;10(2):161. doi: 10.3390/jfmk10020161 (PMC12101270; doi:10.3390/jfmk10020161)
Supplement: Supplementary file 1 [file jfmk-10-00161-s001.zip › jfmk-3500666-supplementary.pdf]

Supplementary Table S1. Results of Linear Mixed-Effects Models for the Experimental Group (RGT): Corrected and Uncorrected p-Values and Significance for Each Clinical Outcome. The table reports the results of linear mixed effects models (LME) for each clinical outcome in the experimental group. For each model, p-values obtained from Wald tests at the term level and their corresponding false discovery rate (FDR)-corrected values are presented, along with the assigned significance symbols. Statistical significance is indicated as ***: $p < 0.001$, **: $p < 0.01$, *: $p < 0.05$ after FDR correction, † indicates results significant before but not after correction, ns denotes non-significant effects.

	**p-value**	**FDR p-value**	**Significance**
**10MWT**			
Age	0.474	0.788	ns
Gender	0.501	0.788	ns
Session	0.202	0.444	ns
TSI	0.756	0.919	ns
Cadence	0.835	0.919	ns
Velocity	0.950	0.950	ns
Age:session	0.039	0.107	†
Gender:session	0.013	0.073	†
TSI:session	0.024	0.089	†
Cadence:session	0.013	0.073	†
Velocity:session	0.623	0.856	ns
**TS Balance**			
Age	0.763	0.839	ns
Gender	0.727	0.839	ns
Session	0.075	0.138	ns
TSI	0.968	0.968	ns
Cadence	0.131	0.181	ns
Velocity	0.020	0.049	*
Age:session	0.099	0.155	ns
Gender:session	0.022	0.049	*
TSI:session	0.004	0.016	*
Cadence:session	0.005	0.016	*
Velocity:session	0.002	0.016	*
**TS Gait**			
Age	0.883	0.971	ns
Gender	0.141	0.311	ns
Session	<0.001	<0.001	***
TSI	0.574	0.749	ns
Cadence	0.179	0.329	ns
Velocity	<0.001	<0.001	***
Age:session	0.510	0.749	ns
Gender:session	<0.001	<0.001	***
TSI:session	0.996	0.996	ns
Cadence:session	0.613	0.749	ns
Velocity:session	<0.001	<0.001	***
**MAS**			
Age	0.208	0.380	ns
Gender	0.002	0.020	*
Session	0.934	0.934	ns
TSI	0.013	0.074	†
Cadence	0.273	0.420	ns

Velocity	0.535	0.617	ns
Age:session	0.089	0.195	ns
Gender:session	0.041	0.143	†
TSI:session	0.306	0.420	ns
Cadence:session	0.052	0.143	ns
Velocity:session	0.561	0.617	ns
**FIM**			
Age	0.665	0.731	ns
Gender	<0.001	0.001	**
Session	0.731	0.731	ns
TSI	<0.001	<0.001	***
Cadence	<0.001	<0.001	***
Velocity	0.015	0.024	*
Age:session	0.013	0.024	*
Gender:session	<0.001	<0.001	***
TSI:session	0.084	0.103	ns
Cadence:session	<0.001	<0.001	***
Velocity:session	0.062	0.085	ns
**FMA Item E**			
Age	0.252	0.461	ns
Gender	0.954	0.954	ns
Session	0.049	0.271	†
TSI	0.124	0.342	ns
Cadence	0.617	0.678	ns
Velocity	0.427	0.522	ns
Age:session	0.037	0.271	†
Gender:session	0.299	0.469	ns
TSI:session	0.251	0.461	ns
Cadence:session	0.404	0.522	ns
Velocity:session	0.088	0.322	ns
**FMA Item F**			
Age	0.222	0.400	ns
Gender	0.312	0.429	ns
Session	0.163	0.359	ns
TSI	0.076	0.329	ns
Cadence	0.102	0.329	ns
Velocity	0.254	0.400	ns
Age:session	0.827	0.874	ns
Gender:session	0.874	0.874	ns
TSI:session	0.120	0.329	ns
Cadence:session	0.740	0.874	ns
Velocity:session	0.104	0.329	ns
**FMA Item E-F**			
Age	0.206	0.410	ns
Gender	0.903	0.903	ns
Session	0.114	0.377	ns
TSI	0.086	0.377	ns
Cadence	0.487	0.595	ns
Velocity	0.691	0.760	ns
Age:session	0.224	0.410	ns
Gender:session	0.454	0.595	ns

TSI:session	0.137	0.377	ns
Cadence:session	0.422	0.595	ns
Velocity:session	0.123	0.377	ns
**FMA Item H**			
Age	0.200	0.314	ns
Gender	0.592	0.723	ns
Session	0.485	0.667	ns
TSI	0.007	0.019	*
Cadence	0.198	0.314	ns
Velocity	<0.001	<0.001	***
Age:session	<0.001	<0.001	***
Gender:session	0.097	0.212	ns
TSI:session	<0.001	<0.001	***
Cadence:session	0.926	0.926	ns
Velocity:session	0.850	0.926	ns
**FMA Item J**			
Age	0.138	0.168	ns
Gender	0.051	0.080	ns
Session	<0.001	<0.001	***
TSI	0.006	0.013	*
Cadence	0.081	0.112	ns
Velocity	0.752	0.752	ns
Age:session	<0.001	<0.001	***
Gender:session	<0.001	<0.001	***
TSI:session	0.004	0.012	*
Cadence:session	0.319	0.351	ns
Velocity:session	0.043	0.079	†
**FMA Item JII**			
Age	0.091	0.162	ns
Gender	0.416	0.436	ns
Session	0.338	0.436	ns
TSI	<0.001	<0.001	***
Cadence	0.005	0.017	*
Velocity	0.044	0.122	†
Age:session	0.436	0.436	ns
Gender:session	0.360	0.436	ns
TSI:session	<0.001	<0.001	***
Cadence:session	0.103	0.162	ns
Velocity:session	0.090	0.162	ns

Legend: FDR (false discovery rate), TSI (time since injury), 10MWT (10 meters walking test), TS Balance (Tinetti balance scale), TS Gait (Tinetti gait scale), MAS (modified Ashworth scale), FIM (functional independence measure), FMA (Fugl-Meyer assessment), Item E (lower extremity), Item F (coordination/speed), Item total E-F (motor function), Item H (sensation), Item JI (passive joint motion), Item JII (joint pain).

Supplementary Table S2. Results of linear mixed effects models for the control group (CGT): corrected and uncorrected p-values and significance for each clinical outcome. The table reports the results of linear mixed effects models (LME) for each clinical outcome in the experimental group. For each model, p-values obtained from Wald tests at the term level and their corresponding false discovery rate (FDR)-corrected values are presented, along with the assigned significance symbols. Statistical significance is indicated as ***: p < 0.001, **: p < 0.01, *: p < 0.05 after FDR correction, † indicates results significant before but not after correction, ns denotes non-significant effects.

	p-value	FDR p-value	Significance
**10MWT**			
Age	0.724	0.767	ns
Gender	0.295	0.460	ns
Session	0.307	0.460	ns
TSI	0.257	0.460	ns
Age:session	0.767	0.767	ns
Gender:session	0.730	0.767	ns
TSI:session	0.121	0.363	ns
**TS Balance**			
Age	0.662	0.745	ns
Gender	0.311	0.400	ns
Session	0.086	0.189	ns
TSI	0.914	0.914	ns
Age:session	0.077	0.189	ns
Gender:session	0.182	0.272	ns
TSI:session	0.105	0.189	ns
**TS Gait**			
Age	0.088	0.113	ns
Gender	0.440	0.440	ns
Session	0.318	0.357	ns
TSI	0.062	0.093	ns
Age:session	0.006	0.019	*
Gender:session	0.061	0.093	ns
TSI:session	0.011	0.024	*
**MAS**			
Age	0.170	0.382	ns
Gender	0.769	0.887	ns
Session	0.799	0.887	ns
TSI	0.887	0.887	ns
Age:session	0.667	0.887	ns
Gender:session	0.628	0.887	ns
TSI:session	0.108	0.325	ns
**FIM**			
Age	0.109	0.139	ns
Gender	0.857	0.857	ns
Session	0.010	0.023	*
TSI	0.010	0.023	*
Age:session	0.635	0.714	ns
Gender:session	0.036	0.065	†
TSI:session	0.086	0.130	ns

Legend: FDR (false discovery rate), TSI (time since injury), 10MWT (10 Meters Walking Test), TS Balance (Tinetti balance scale), TS Gait (Tinetti gait scale), MAS (Modified Ashworth Scale), FIM (Functional Independence Measure).
